# Interplay of Sphingolipid Metabolism in Predicting Prognosis of GBM Patients: Towards Precision Immunotherapy

**DOI:** 10.7150/jca.89338

**Published:** 2024-01-01

**Authors:** Qi Wang, Chuanhua Zheng, Hanjin Hou, Xin Bao, Huading Tai, Xufeng Huang, Zhengrui Li, Zhangzuo Li, Qiaowei Wang, Qi Pan, Longbin Wang, Shujing Zhou, Yanjie Bian, Qier Pan, Aihua Gong, Min Xu

**Affiliations:** 1 Department of Gastroenterology, Affiliated Hospital of Jiangsu University, Jiangsu University, Zhenjiang, China.; 2 Department of Neurosurgery, the Affiliated Hospital of Youjiang Medical University for Nationalities, Guangxi, China.; 3 Department of Cell Biology, School of Medicine, Jiangsu University, Zhenjiang, China.; 4 School of Clinical Medicine, Guizhou Medical University, Guiyang, China.; 5 Faculty of Dentistry, University of Debrecen, Debrecen, Hungary.; 6 Department of Oral and Maxillofacial-Head and Neck Oncology, Shanghai Ninth People's Hospital, Shanghai Jiao Tong University School of Medicine, College of Stomatology, Shanghai Jiao Tong University, Shanghai, China.; 7 National Center for Stomatology & National Clinical Research Center for Oral Diseases, Shanghai, China.; 8 Shanghai Key Laboratory of Stomatology, Shanghai, China.; 9 Department of Clinical Veterinary Medicine, Huazhong Agricultural University, Wuhan, China.; 10 Faculty of Medicine, University of Debrecen, Debrecen, Hungary.; 11 Xinxiang Medical University, Xinxiang, China.; 12 Department of Endocrinology, Affiliated Hospital of Jiangsu University, Jiangsu University, Zhenjiang, China.

**Keywords:** Sphingolipid, lncRNAs, GBM, Precision immunotherapy, Biomarkers

## Abstract

**Background:** In spite of numerous existing bio-surveillance systems for predicting glioma (GBM) prognosis, enhancing the efficacy of immunotherapy remains an ongoing conundrum. The continual scrutiny of the dynamic interplay between the sphingolipid metabolic pathway and tumor immunophenotypes has unveiled potential implications. However, the intricate orchestration of functional and regulatory mechanisms by long non-coding RNAs (lncRNAs) in GBM, particularly in the context of sphingolipid metabolism, remains cryptic.

**Methods:** We harnessed established R packages to intersect gene expression profiles of GBM patients within the The Cancer Genome Atlas (TCGA) database with the compilation of sphingolipid metabolism genes from GeneCards. This enabled us to discern markedly distinct lncRNAs, which were subsequently deployed to construct a robust prognostic model utilizing Lasso-Cox regression analysis. We then scrutinized the immune microenvironment across various risk strata using the ssGSEA and CIBERSORT algorithms. To evaluate mutation patterns and drug resistance profiles within patient subgroups, we devised the "Prophytic" and "Maftools" packages, respectively.

**Results:** Our investigation scrutinized lncRNAs linked to sphingolipid metabolism, utilizing glioma specimens from TCGA. We meticulously curated 1224 sphingolipid-associated genes gleaned from GeneCards and pinpointed 272 differentially expressed mRNAs via transcriptomic analysis. Enrichment analyses underscored their significance in sphingolipid processes. A prognostic model founded on 17 meticulously selected lncRNAs was systematically constructed and validated. This model adeptly stratified GBM patients into high- and low-risk categories, yielding highly precise prognostic insights. We also discerned correlations between immune cell infiltration and genetic mutation discrepancies, along with distinct therapeutic responses through drug sensitivity analysis. Notably, computational findings were corroborated through experimental validation by RT-PCR.

**Conclusion:** In summation, our exhaustive inquiry underscores the multifaceted utility of the sphingolipid metabolic pathway as an autonomous diagnostic and prognostic indicator for glioma patients. Furthermore, we amalgamate a profusion of substantiated evidence concerning immune infiltration and gene mutations, thereby reinforcing the proposition that sphingolipid metabolism may function as a pivotal determinant in the panorama of immunotherapeutic interventions.

## Introduction

Glioma, a profoundly invasive and heterogeneous neuroepithelial neoplasm originating from glial cells within the encephalon and central nervous system, poses a formidable quandary in clinical settings 1. It predominantly afflicts adults, reaching its zenith between the ages of 31 and 40, and represents the most prevalent malignancy of the cerebral hemisphere. The clinical manifestations of glioma encompass a wide spectrum of symptoms, encompassing cephalalgia, emesis, visual and linguistic impairments, and seizures 2. Gliomas can be categorized into diverse subtypes, including astrocytoma, glioblastoma, and oligodendroglioma, based on cellular differentiation. In 2007, the World Health Organization (WHO) standardized the classification of gliomas into Grades I to IV. Glioblastoma (GBM), a Grade IV astrocytoma, epitomizes the most malignant and daunting primary intracranial neoplasm 3, 4. Histologically, it is characterized as a diffusely infiltrating glioma originating from astrocytic lineage. GBM accounts for approximately 50% of all gliomas and exhibits an overall bleak prognosis, positioning it as one of the most lethal intracranial neoplasms 3, 5. The precise etiological mechanisms underlying GBM pathogenesis remain incompletely elucidated, however, investigations have implicated a multitude of factors, encompassing genetic mutations, epigenetic modifications, environmental influences, and dysregulation of intracellular and extracellular signaling pathways. Pertinent genetic alterations encompass mutations in the tumor suppressor genes TP53 and PTEN, as well as oncogenic activation of EGFR, all of which have emerged as pivotal contributors to the development of GBM 5. Currently, GBM lacks efficacious therapeutic strategies, and the standard therapeutic approach entails multimodal interventions aimed at protracting patient survival. This typically encompasses surgical resection followed by radiotherapy and chemotherapy, incorporating the administration of temozolomide (TMZ), which collectively constitutes the Stupp regimen 6, 7. In spite of notable progress in the management of GBM in recent decades, the prognosis remains disheartening. GBM is characterized by infiltrative growth patterns, resulting in both intratumoral and extratumoral metastatic lesions 8. Its responsiveness to therapy is limited, and it exhibits a high recurrence rate. Currently, there are no curative approaches for GBM. Metabolomics, an all-encompassing scientific field involving the systematic analysis and identification of metabolites within an organism using high-throughput methods, seeks to unveil the metabolic profiles of biological systems and their connections to health and disease. Metabolomics has gained increasing recognition and has found various applications in clinical practice, particularly in deciphering the complexities of GBM progression through rigorous clinical investigations 9, 10.

Sphingolipids also referred to as cerebrosides, comprise a group of lipid molecules that exhibit wide distribution and significant conservation within the central nervous system 11. These molecules, predominantly constituted by ceramides, sphingomyelins (SM), and glycosphingolipids, represent major lipid components in eukaryotic organisms 12. They possess a distinct lipid framework consisting of sphingosine, an amino alcohol, and a fatty acid residue. Sphingolipids have demonstrated pivotal involvement in diverse cellular functions 13. Glycosphingolipids, highly expressed in the brain, serve as integral constituents of cell membranes 13. Additionally, they constitute the primary lipid constituents of the myelin sheath enveloping neuronal axons, playing a critical role in cellular signaling 14. Ceramides are recognized regulators of target proteins such as protein kinase D (PKD) and p53, eliciting pro-apoptotic or cellular senescence effects 15, 16. Moreover, ceramides have been shown to induce cell apoptosis by facilitating the formation of mitochondrial outer membrane pores and the subsequent release of cytochrome C 17. Furthermore, sphingosine and ceramide assume essential roles in various fundamental cellular physiological and biochemical processes, encompassing cell apoptosis and autophagy, regulation of inflammation, maintenance of vascular endothelial integrity, modulation of stress response, and regulation of angiogenesis, smooth muscle contraction, and relaxation 11. Sphingolipids may likewise profoundly impact the immune microenvironment of tumors. Specifically, these processes could encompass the infiltration, differentiation, and downstream cascade effects exerted by immune cells within the tumor microenvironment (TME). Conceivably, these processes could influence tumor cell proliferation, survival, migration, angiogenesis, invasion, and the response to chemotherapeutic and immunotherapeutic agents 18, 19. As mentioned above, the feasibility of sphingolipids, as a class of active substances prominently present in the nervous ecosystem, whether being able to act as an integral part in the formation of neurological tumours, deserves to be questioned and is also of considerable research value 20, 21. Despite the availability of an extensive range of therapies for glioma treatment, the effectiveness of current treatments is considerably suboptimal. Investigating the involvement of sphingolipid metabolism in glioma has the potential to provide new insights and avenues for the exploration of innovative approaches to glioma therapy.

**Figure 1:**
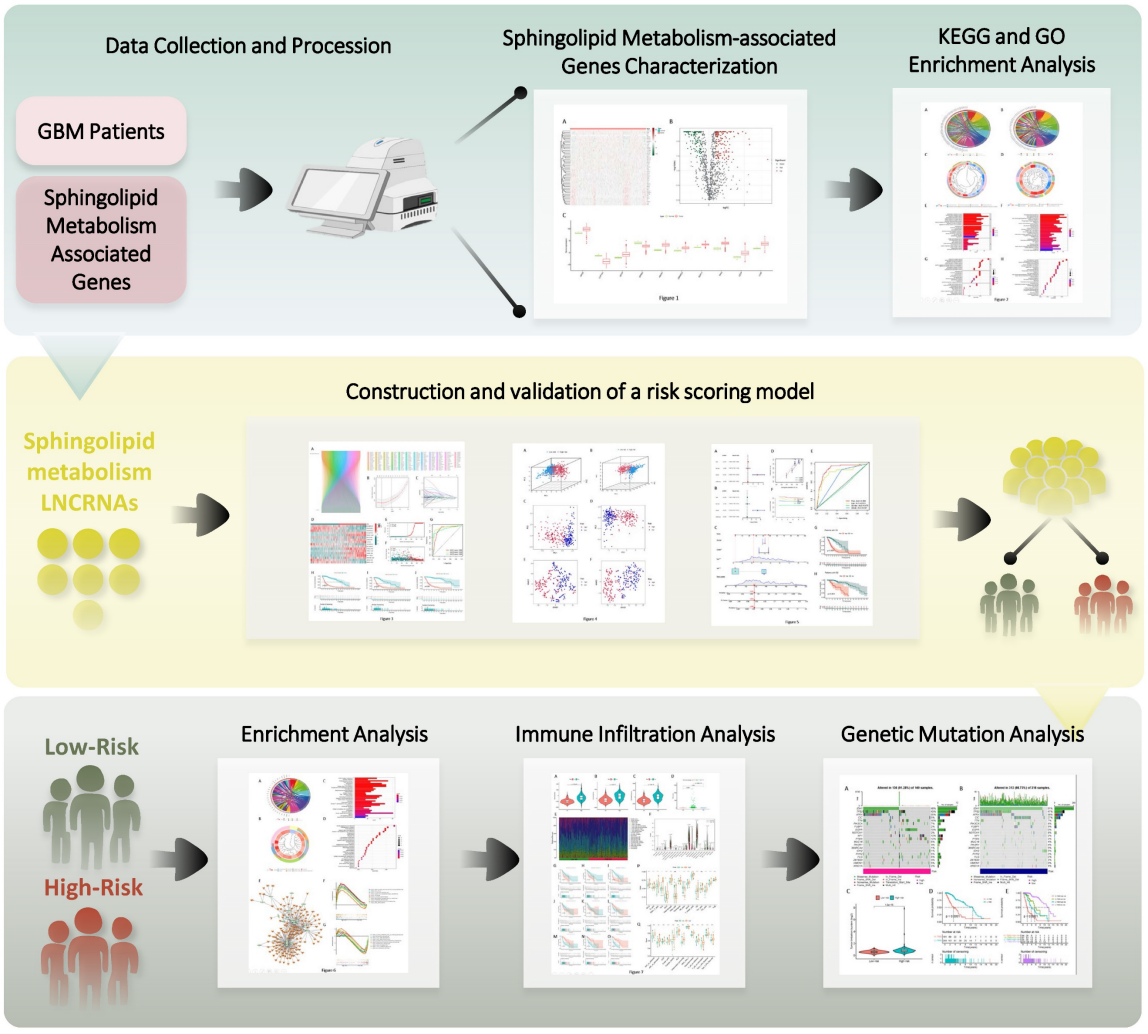
Outline of the research program.

Given the rapid evolution of bioinformatics technology, the tangible utility of sphingolipid metabolism in glioma and its associated mechanisms within existing data resources can be thoroughly explored 22. To this end, we established a connection between the sphingolipid metabolic pathway and GBM using the available transcriptomic data. The genetic model of the sphingolipid metabolic pathway aptly reflected patient prognosis, while also offering valuable insights into immune infiltration and gene mutation profiles, thereby presenting a fresh methodology for achieving even greater precision in GBM chemotherapy and immunotherapy (Figure 1).

## Materials and Methods

### Access to information on GBM patients

We obtained RNA sequencing datasets and relevant clinical characteristics of glioblastoma multiforme (GBM) patients from The Cancer Genome Atlas (TCGA). A total of 44 normal tissues and 504 GBM cases were collected. Our inclusion criteria entailed comprehensive expression data for long non-coding RNAs (lncRNAs), clinical information, and a minimum 30-day follow-up period for GBM patients. Since we utilized pre-existing data from a publicly available database, ethical approval was not necessary for this study.

### Selection of Sphingolipid Metabolism-Associated LncRNAs

We retrieved sphingolipid-related genes directly from the GeneCards database (https://www.genecards.org/). Within this database, we selected genes associated with sphingolipids that exhibited a correlation score exceeding 10. TCGA provided lncRNA profiles of patients, and after normalizing the data, we employed the limma software package for further analysis. R software facilitated Pearson correlation studies between lncRNAs and relevant genes in GBM patients. Cytoscape was utilized to construct co-expression networks linking sphingolipid-related lncRNAs and corresponding genes 23.

### Development of Risk Scores

To identify lncRNAs associated with sphingolipid-related genes and their impact on the survival of GBM patients, we conducted univariate Cox proportional hazard regression analysis on clinical data obtained from TCGA. Our analysis revealed statistically significant associations (p<0.05) between lncRNAs and survival outcomes. To refine the selection of relevant lncRNAs, we performed 10-fold cross-validation using LASSO regression with a significance threshold of 0.05, iterating the process 1000 times to mitigate overfitting. Additionally, we employed 1000 instances of random stimulation. Through multivariate Cox regression analysis, we selected target genes and established an independent prognostic feature. We computed risk scores for each patient sample by multiplying the expression levels of each lncRNA by their respective weights, as defined in the multivariate Cox model.

### Validation of Predictive Markers

Clinical data underwent univariate and multivariate Cox regression analysis to assess whether the risk score could serve as an independent prognostic indicator. Furthermore, c-index and ROC curve analysis were employed to verify the association between prognostic factors and the risk score, thereby validating the predictive biomarkers.

### Construction and Application of Nomination Map

To determine the consistency between predictive results and observed data, we utilized the rms R package to construct a nomination plot and calibration curve. This involved considering various factors such as risk score, age, gender, grade, tumor size (T), lymph node metastasis (N), and tumor stage. Additionally, we employed Kaplan-Meier plot analysis to validate the predictive efficacy of the nomination plot across different clinical stages.

### Functional Enrichment Analysis

To evaluate functional enrichment, we utilized the Kyoto Encyclopedia of Genes and Genomes (KEGG), Gene Ontology (GO), and Genome Set Enrichment Analysis (GSEA).

### Analysis of Immune Cell Infiltration

To determine the relationship between lncRNA characteristics and immune cell infiltration, we obtained a gene-expression matrix dataset by filtering and presenting the data using CIBERSORT 24. This method allowed us to deduce the relative proportions of 22 immune cell populations in each supplemented sample. Comparisons were made to identify differences in infiltrating immune cell populations between high- and low-risk groups. Furthermore, we applied the ssGSEA algorithm, representing Genomic Variance Analysis (GSVA) with default parameters, to estimate the degree of infiltration of different immune cell types outside of the sample. The ssGSEA algorithm, a rank-based method, computes a grade indicating the absolute enrichment of the genome by incorporating genomes from other publications 25.

### Comparison of Mutation Information among Subgroups

We utilized the "maftools" software package to profile mutations in high- and low-risk populations, determine the tumor mutational burden (TMB) for each patient, generate waterfall plots, and explore the relationship between the risk score of the prediction model and mutated elements such as IDH1 in the TCGA dataset 26.

### Drug Sensitivity Assessment

To assess the role of established genetic markers associated with sphingolipid metabolism in response to different chemotherapeutic and immunotherapeutic drugs, we calculated the IC50 values of various drugs for GBM patients based on the Genomics of Drug Sensitivity in Cancer (GDSC) database using the "prophytic" package. Subsequently, sensitivity predictions were performed.

### Cell culturing

All cell lines authenticated by the STR method for human glioma cells NHA, U87, LN229, and LN18 were purchased from the Cell Bank of the Chinese Academy of Sciences. Cultivation of cells was performed in a culture system of 10% fetal bovine serum mixed with RPMI1640, and the overall environment was maintained in a sterile incubator at a constant temperature of 37°C.

### RT-PCR

Manufacturer-recommended RNAiso-Plus (Takara) was preferred for total RNA isolation and extraction, which was then reverse-transcribed into cDNA with the support of a high-capacity gene synthesis kit (Takara, China). Referring to the standards provided by the reagent supplier, the optimal conditions for the reaction were determined, GAPDH was adopted as an internal reference for abundance assay, and the relative expression was determined by the 2-ΔΔΔCt method. Respectively, the relative expression was calculated using the 2-ΔΔCt method.

## Results

### Characterization of lncRNAs Associated with Sphingolipid Metabolism through Transcriptomic Analysis

**Figure 2:**
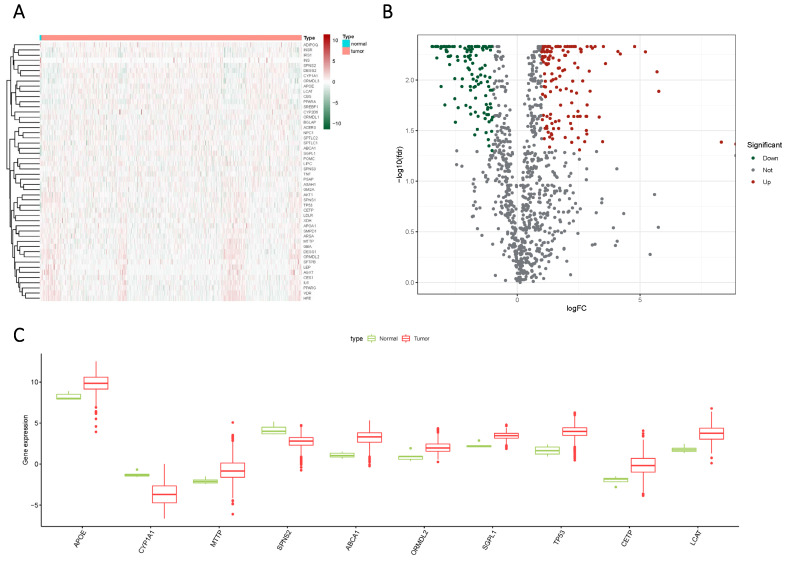
The expression of sphingolipid metabolism-related genes was compared between GBM and normal subjects using different visualization techniques. (A) Expression levels of these genes were represented through a Heatmap across the two samples. (B) A Volcano plot was used to depict the differential expression of genes associated with sphingolipid metabolism in both samples. (C) A Cricket diagram was used to illustrate the differential expression of genes involved in sphingolipid metabolism between the two samples.

**Figure 3:**
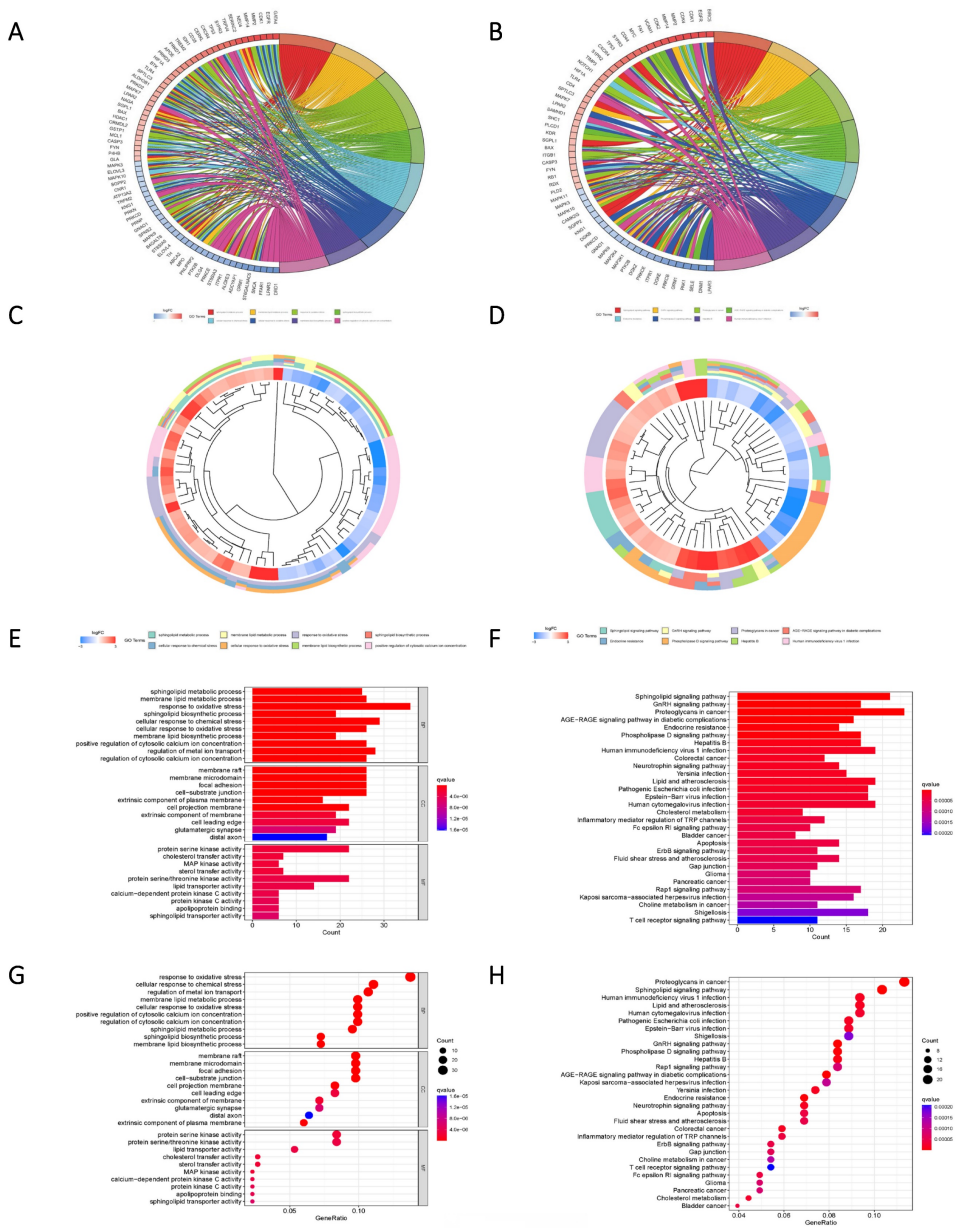
Pathway enrichment analysis based on Differential genes. (A, C, E, G) The predicted results of the KEGG pathway for the differential genes. (B, D, F, H) GO pathway prediction results of differential genes.

Glioma samples, comprising both normal and tumor tissues, were acquired from the TCGA dataset. A total of 1224 genes linked to sphingolipid metabolism were extracted from the GeneCards website. To visually illustrate the disparities between normal and tumor samples, a randomized subset of 50 genes was depicted in a heatmap (Figure 2 A). By scrutinizing the expression patterns of 1020 genes associated with sphingolipid metabolism (with a correlation score >10) across the samples, we discerned a total of 272 differentially expressed mRNAs (Figure 2 B, C). Enrichment analysis based on KEGG and GO pathways revealed noteworthy enrichment in processes such as Sphingolipid metabolic process, Membrane lipid metabolic process, Sphingolipid signaling pathway, and GnRH signaling pathway (Figure 3).

### Construction and Validation of a Risk Scoring Model for the Prognostic Assessment of lncRNAs Associated with Sphingolipid Metabolism

**Figure 4:**
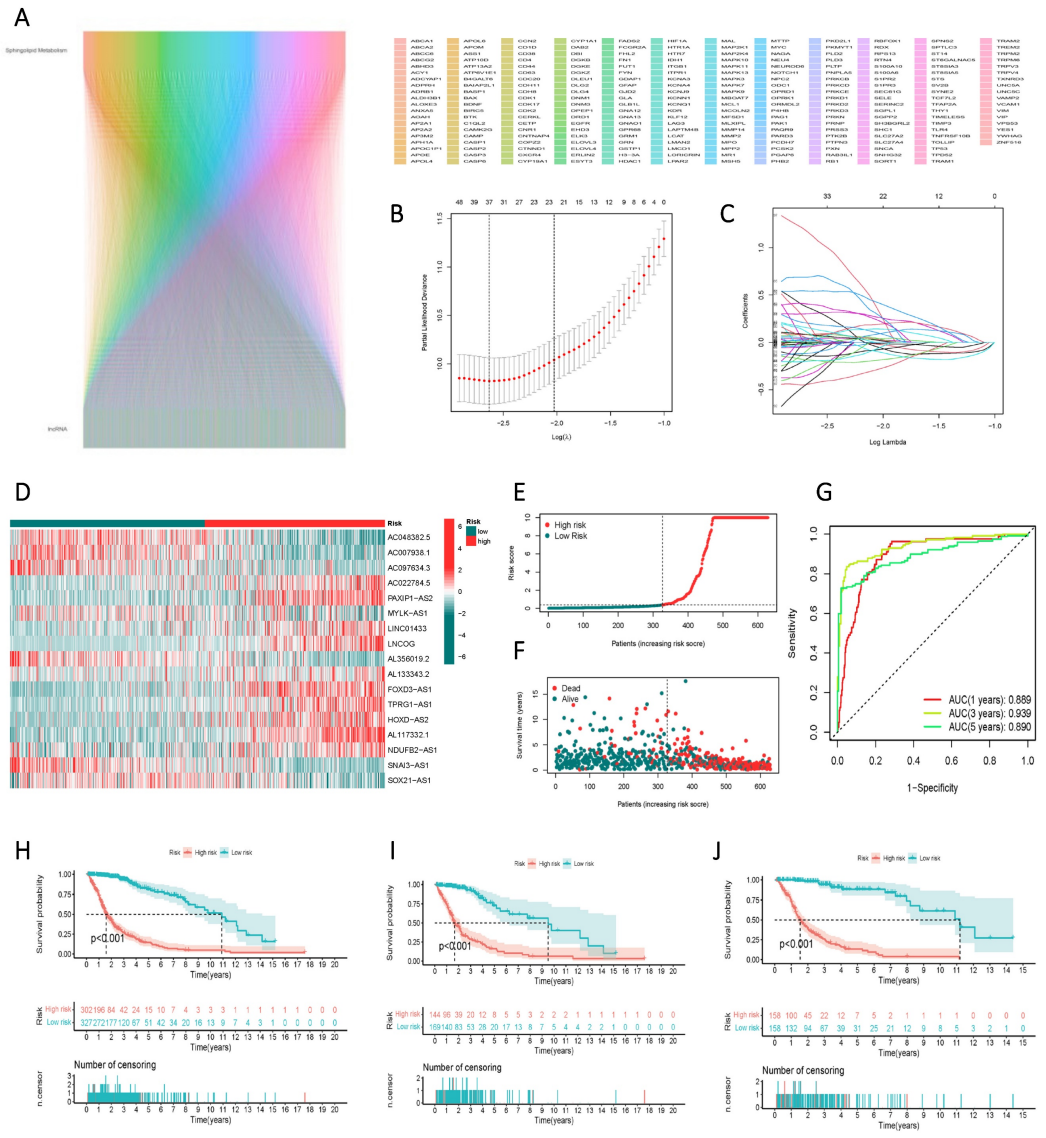
Identification of lncRNAs associated with sphingolipid metabolism and their prognostic significance in GBM. (A) Prognostic lncRNAs identified by univariate Cox proportional hazards regression analysis. (B, C) The LASSO-Cox regression model was used to select lncRNAs associated with sphingolipid metabolism. (D) The heat map depicts the expression of 24 lncRNAs in both high-risk to low-risk cohorts. (E, F) Riskscore, survival condition, and survival duration of GBM recipients were distributed. (G) ROC curves show the predictive efficacy of risk scores on patient survival at 1, 3, and 5 years. (H, I, J) Kaplan-Meier profiles reflect the survival state or survival duration in higher and lower-risk categories of GBM patients.

Through correlation analysis, we integrated lncRNAs associated with sphingolipid metabolism that met the criterion of R > 0.4 and p < 0.001 into the gene screening process to construct the risk model (Figure 4 A). Ultimately, 17 genes were selected using univariate Cox regression, LASSO regression (Figure 4 B, C), and multivariate Cox regression analyses to establish the risk scores. The 17 variables employed in the model construction comprised AC048382.5, AC007938.1, AC097634.3, AC022784.5, PAXIP1-AS2, MYLK-AS1, LINC01433, LNCOG, AL356019.2, AL133343.2, FOXD3-AS1, TPRG1-AS1, HOXD-AS2, AL117332.1, NDUFB2-AS1, SNAI3-AS1, and SOX21-AS1 (Figure 4 D). Based on the median risk score, GBM patients from the TCGA study were categorized into high-risk or low-risk groups. The Kaplan-Meier curve exhibited significant divergence in overall survival (OS) between the two groups, with the high-risk group displaying poorer OS (Figure 4 E). ROC curve analysis demonstrated the risk score's efficacy in differentiating outcomes for GBM patients at 1, 3, and 5 years, yielding AUC values of 0.889, 0.939, and 0.890, respectively (Figure 4 F). The risk score facilitated the classification of GBM patients into distinct risk classes (Figure 4 G), and scatter plots depicting the correlation between risk score and survival rate, illustrating increased mortality with higher risk scores (Figure 4 H, I, J).

**Figure 5:**
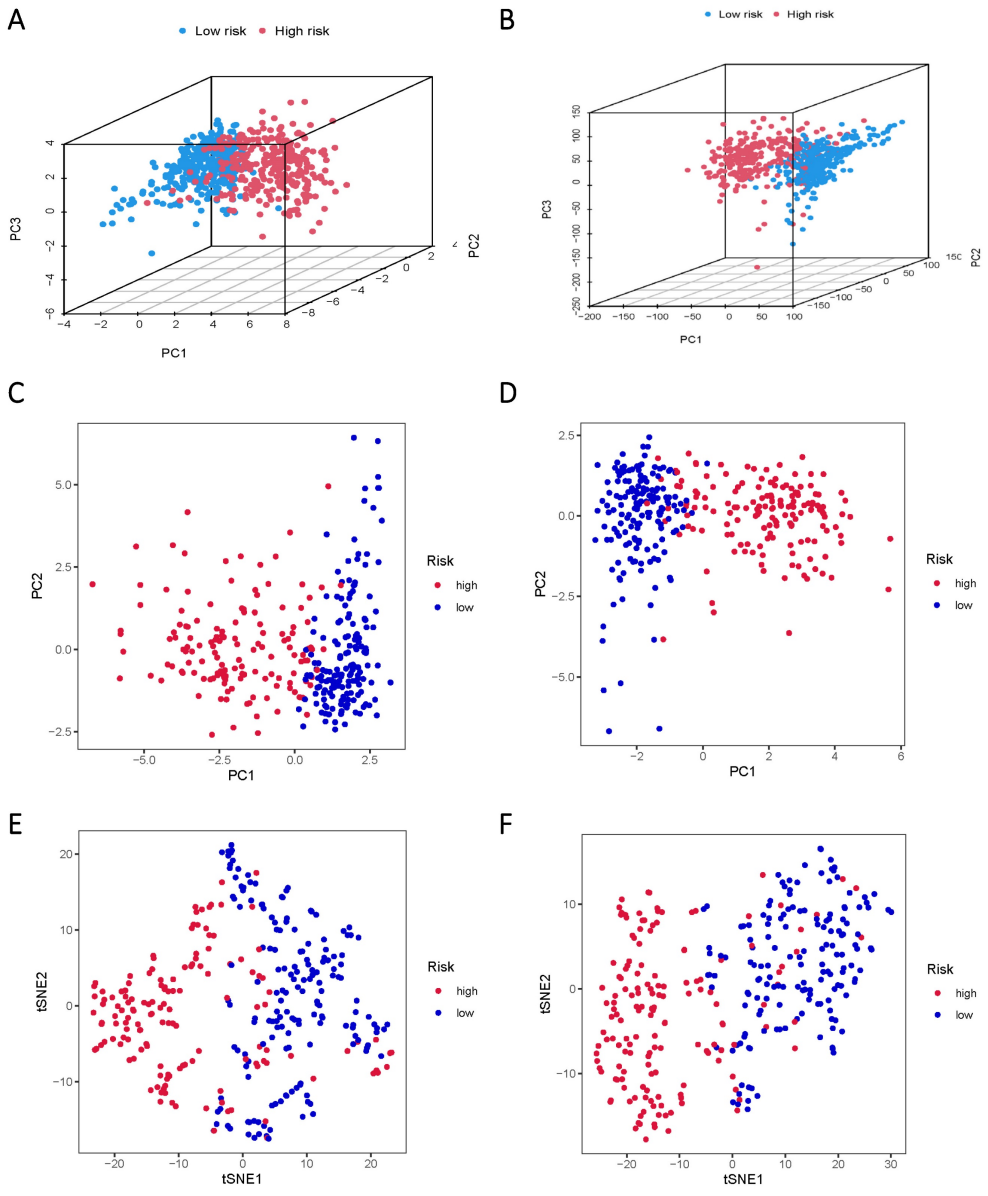
PCA and t-SNE plots are analyzed between low and high-risk groups based on the expression profiles of 17 sphingolipid metabolism-related lncRNAs. (A, B) PCA-3D plot analysis of the training and test groups. (C, D) PCA plot analysis between the training and test groups. (E, F) tSNE plot analysis of the training and test groups.

The TCGA data was subsequently divided into training and validation groups based on the risk model, affirming its significant prognostic value. Principal Component Analysis (PCA) and t-distributed Stochastic Neighbor Embedding (t-SNE) analyses were performed to elucidate the expression disparities between the low-risk and high-risk groups in both training and test cohorts (Figure 5A-F). Clustering analysis validated the ability of risk scores based on the 17 lncRNAs associated with sphingolipid metabolism to effectively discriminate between high-risk and low-risk patients in both groups.

### Evaluation of the Prognostic Model's Efficacy in lncRNAs Involved in Sphingolipid Metabolism

**Figure 6:**
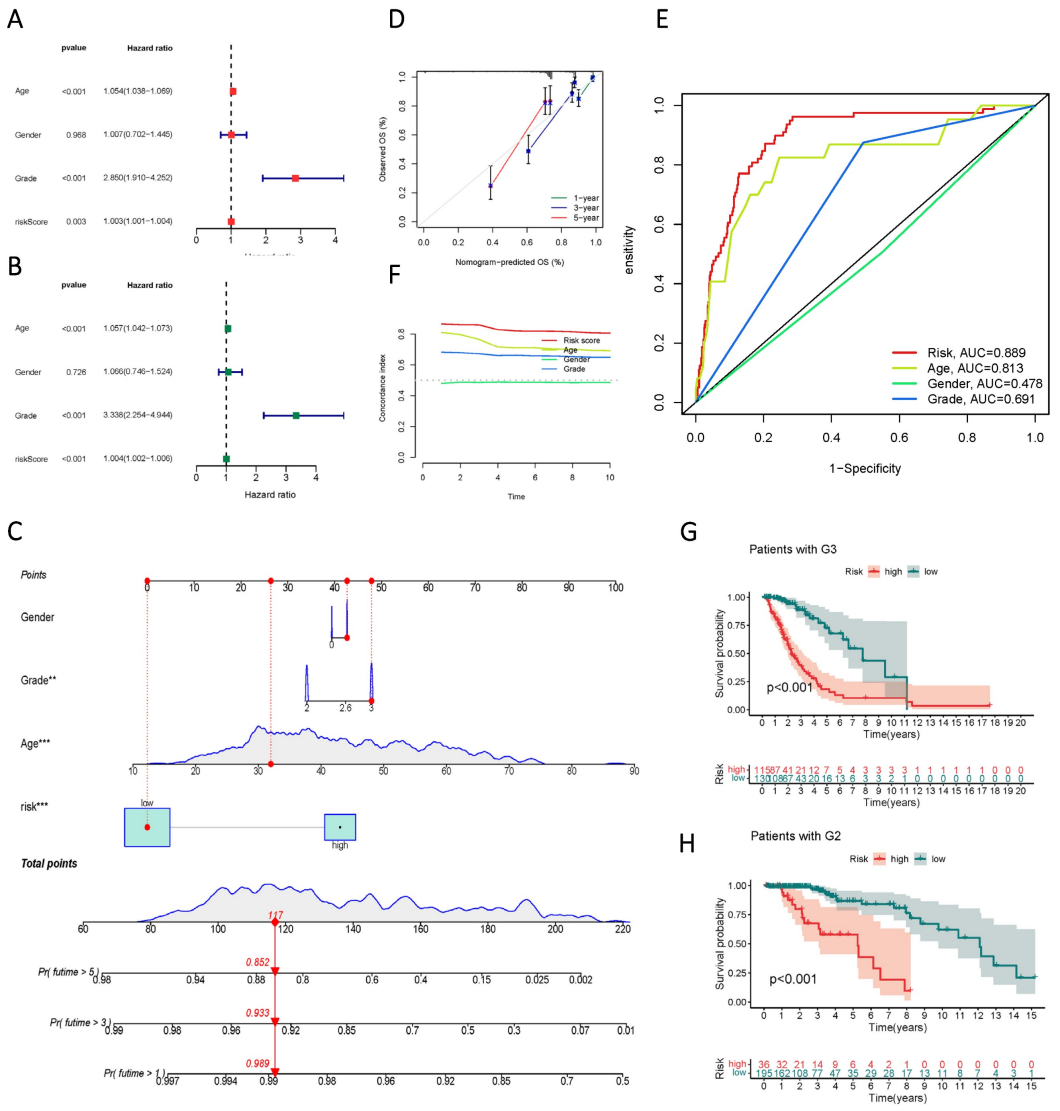
Validity assessment of sphingolipid metabolism lncRNAs to construct prognostic models. (A, B) Univariate and multivariate Cox regression analysis reveals the prognostic value of current risk scores and clinical characteristics. (C) Columnar line graph models are constructed based on risk score, age, gender, and tumor stage. (D) A calibrated plot of overall patient survival at 1 year, 3 years, and 5 years. (E) ROC curves of risk scores and clinical features predicting prognosis. (F) C-index for risk score, grade, age, and gender. (G, H) Kaplan-Meier curves for subgroup analysis based on tumor stage.

Univariate and multivariate Cox regression analyses identified age, gender, staging, and risk score as independent risk factors (HR > 1, p < 0.05) (Figure 6 A, B). Column line plots were generated to visualize the prognostic information of risk score, age, gender, and staging (Figure 6 C, D, F). Calibration plots at 1, 3, and 5 years accurately predicted overall survival (OS) using the prognostic model (Figure 6 E). The C-index and ROC analysis revealed the robust prognostic ability of the risk score, with an AUC (Risk Score) of 0.889. Moreover, the line graphs effectively differentiated patients at different clinical stages, as exemplified by G2 and G3 patients (Figure 6 G, H).

### KEGG and GO enrichment analysis of high- and low-risk groups according to Risk Score

**Figure 7:**
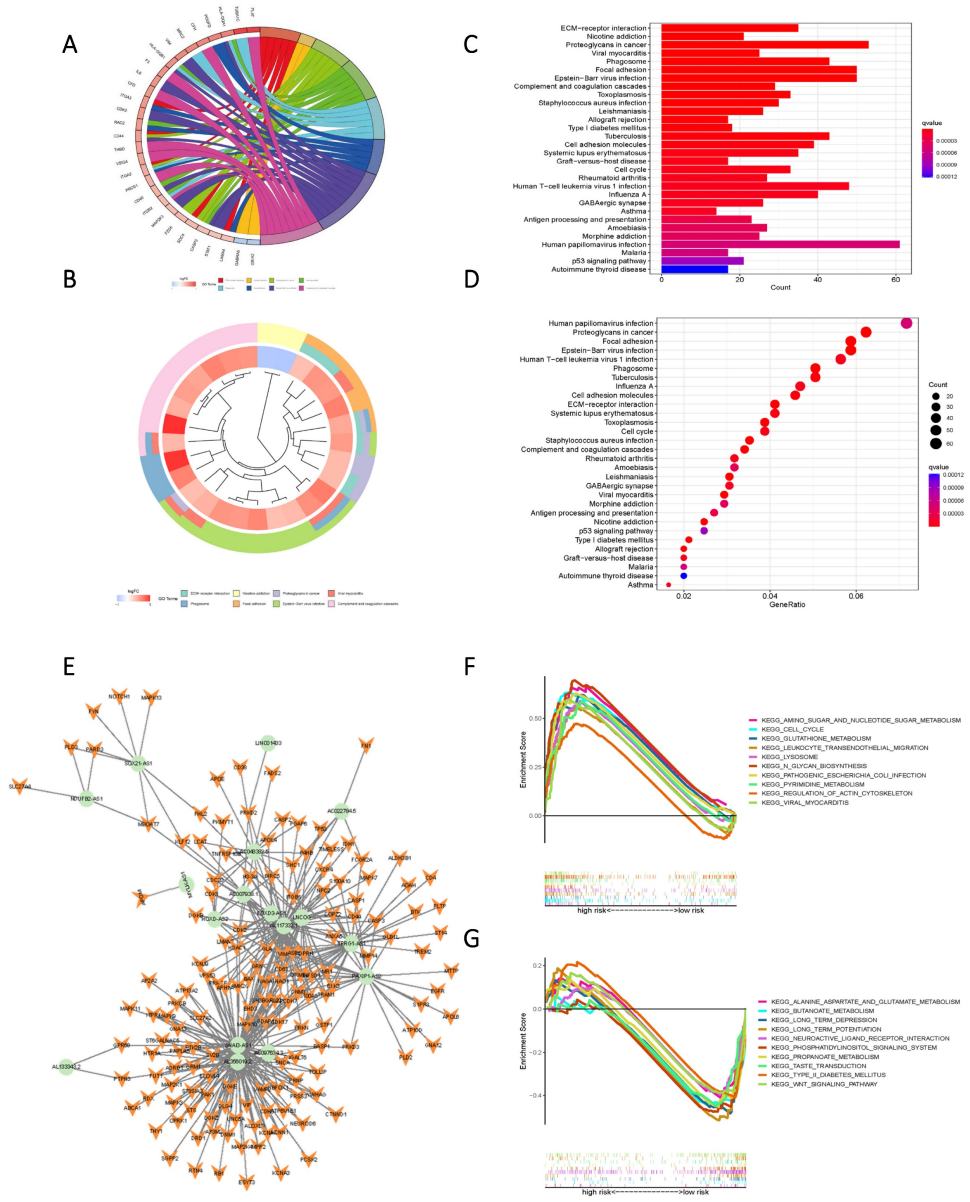
High and low-risk groups in the pathway enrichment analysis. (A, B, C, D) KEGG analysis of DEGs in two groups with high and low risk. (E) LncRNAs-mRNAs co-expression network of GBM. (F) GSEA results reveal significant enrichment of the top 10 pathways in the high-risk group. (G) Top 10 pathways significantly enriched in the low-risk group by GSEA analysis

KEGG and GO enrichment analysis based on Risk Scores for cohorts with varying levels of risk yielded subsequent findings that highlighted significant functional disparities. These distinctions were predominantly manifested in pathways encompassing ECM-receptor interaction, Nicotine addiction, Proteoglycans in cancer, Viral myocarditis, Phagosome, Focal adhesion, Human papillomavirus infection, Proteoglycans in cancer, and Epstein-Barr virus infection (Figure 7 A-D). To further elucidate the nuanced functional variability of the existing prognostic models within diverse risk populations, we established a co-expression network that integrated risk scores and 17 lncRNAs associated with oxidative lipid metabolism (Figure 7 E). The utilization of GSEA software facilitated a comprehensive examination of the distinct pathway information associated with the current prognostic model. Notably, high-risk populations demonstrated enrichment in Amino sugar and nucleotide sugar metabolism, as well as Pyrimidine metabolism and Glutathione metabolism. Conversely, the low-risk group exhibited enriched pathways in Alanine aspartate and glutamate metabolism, Butanoate metabolism, and Propanoate metabolism (Figure 7 F, G). Consequently, the present Risk Score effectively discerns between high and low-risk groups.

### Analysis of immune cell infiltration

**Figure 8:**
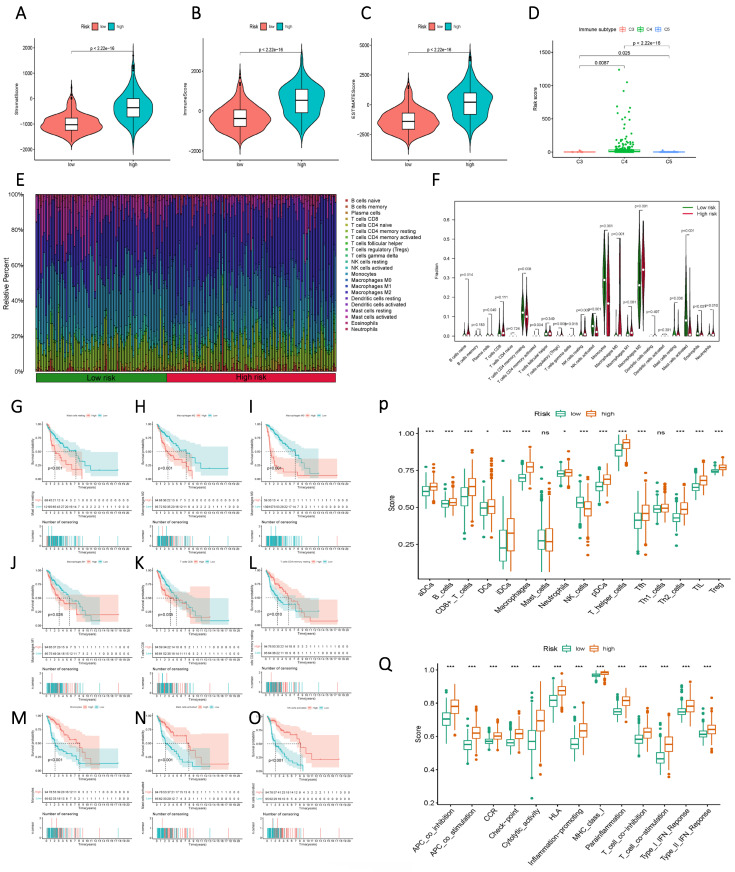
Immunological characteristics differences between high and low-risk subgroups based on Risk Score. (A) StromalScore of the two risk groups. (B) ImmuneScore of the two risk groups. (C) ESTIMATEScore of the two risk groups. (D) Variation in Risk Score of the different immune subgroups. (E) a histogram showing the proportion of immune cells in each of the two groups; (F) a violin plot indicating the difference in the levels of immune cell infiltration between the two groups; (G-O) Kaplan-Meier curves illustrating the association between immune infiltrating cells and prognosis; (P, Q) a violin plot displaying the differences in immune cell and immune function scores between the two groups.

Initially, a comparison of StromalScore, ImmuneScore, and ESTIMATEScore between the high and low-risk groups, based on Risk Score, unveiled a significant divergence, with the high-risk group exhibiting elevated Risk Scores (Figure 8 A-C). This contrast extended to distinct immune subgroups, indicating a close association between the current sphingolipid metabolism model and immune function (Figure 8 D). Further meticulous analysis of immune function was undertaken through histograms that illustrated the composition of 22 immune cells, as well as violin plots that demonstrated variations between the high and low-risk groups across discrete immune cell populations (Figure 8 E, F). Integration of prognostic information revealed that higher proportions of mast cells, macrophages, and CD8 T cells were indicative of an unfavorable prognosis for patients (Figure 8 G-O). Conversely, higher proportions of CD4 T cells and NK cells were associated with a favorable prognosis. Immune cell and immune function scores exhibited variations between the low and high-risk groups (Figure 8 P, Q). The overall trend aligned with the high and low-risk scores, thereby indicating that subgroups with elevated risk scores were more likely to exhibit advanced functional characteristics.

### Genetic mutation analysis in populations with distinct risk profiles

**Figure 9:**
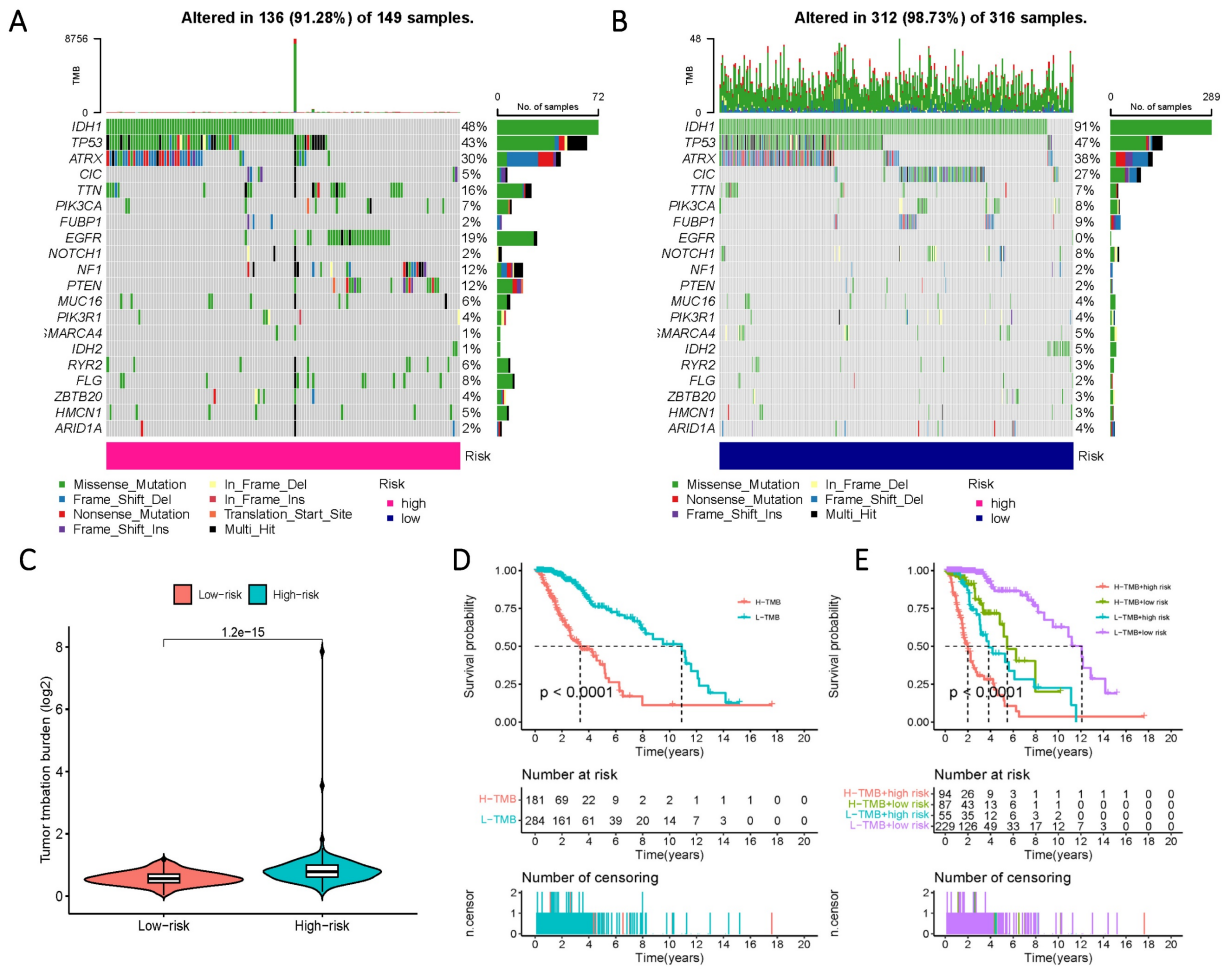
Prognosis of mutation patterns in high- and low-risk populations. (A) Mutation information for high-risk populations. (B) Mutation profile of the low-risk population. (C) TMB in the two risk groups. (D) Prognostic information for patients with high and low TMB scores. (E) Prognostic information for patients with high and low TMB scores in risk groups with differing Risk Score definitions.

To comprehensively characterize gene mutations in the high- and low-risk groups, we examined significant mutations in genes such as IDH1, TP53, ATRX, and CIC, among others. The results yielded striking findings: firstly, the mutation rate of IDH in the low-risk group reached 91%, whereas, in the high-risk group, the mutation rate of IDH1 was less than 50%. Another notable distinction was observed in the mutation rate of EGFR, which amounted to 19% in the high-risk group and 0% in the low-risk group (Figure 9 A, B). Additionally, a significantly higher tumor mutational load was observed in the high-risk group, further reflecting its prognostic impact, as evidenced by a considerable reduction in survival time within the high-TMB group (Figure 9 C-E).

### Identification of potential drugs for diverse subgroups

**Figure 10:**
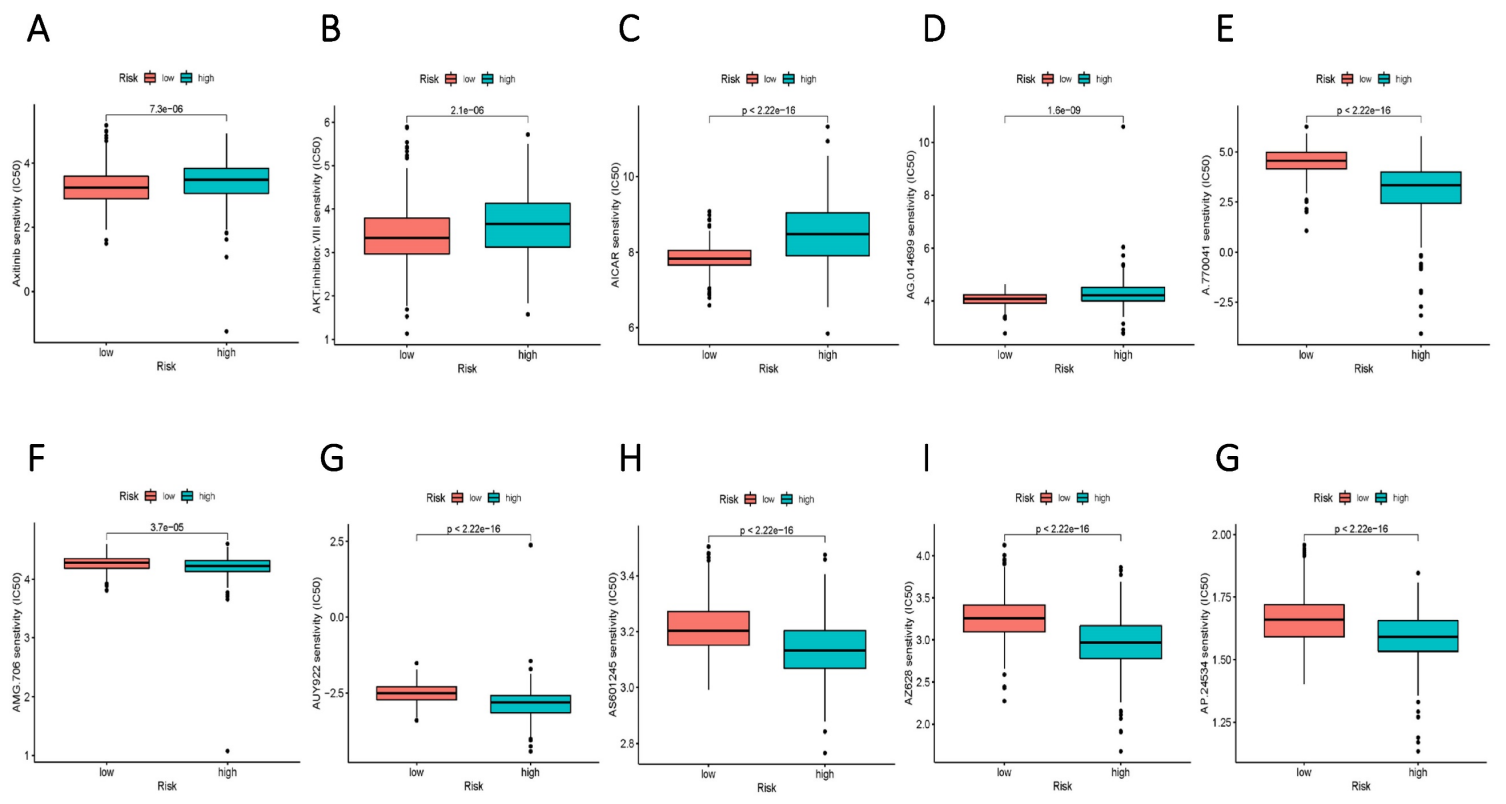
Potential drug filter for high and low-risk groups. (A-E) More sensitive medications for the high-risk group. (F-G) Drugs that are more sensitive to the low-risk group.

To augment the translational value of the current sphingolipid metabolism prognostic model, drug prediction analysis was conducted. We judged by relying on the index of IC50, the lower IC 50 value indicates increased drug sensitivity, and different risk groups showed distinct drug applicability intervals, specifically, the low-risk group was more sensitive to Axitinib, AKT inhibitor, AICAR, and AG.014699. While, the high-risk group presented stronger sensitivity to drugs such as A.770041, AMG.706, AUY922. The above results could more accurately guide precision therapy through the current risk-prognostic model. (Figure 10 A-E).

### RT-PCR validation

**Figure 11:**
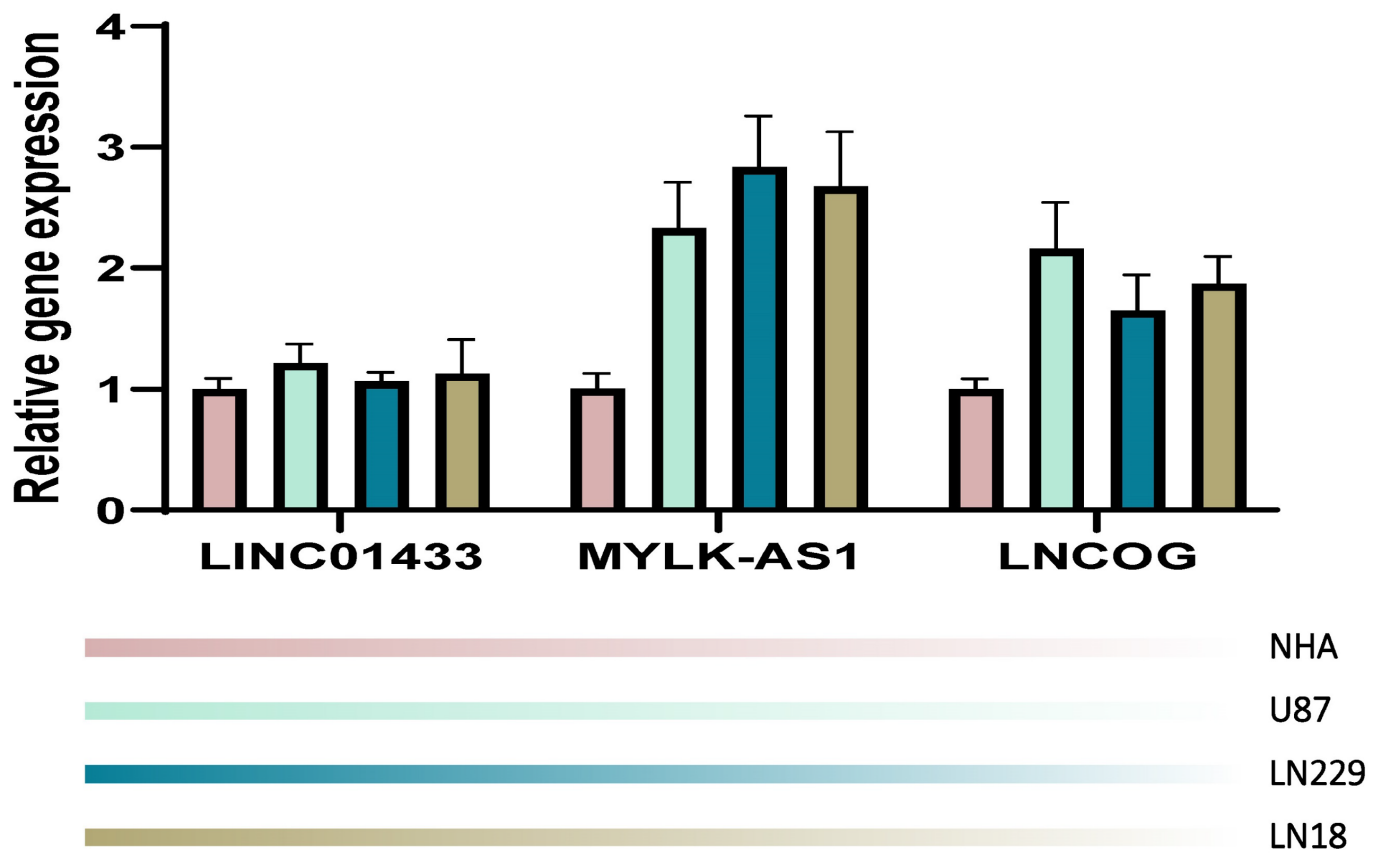
RT-PCR validation. Compared with the NHA cell line, the tumorous cell lines expressed LINC01433, MYLK-AS1, and LNCOG in a higher level.

We validated our computation in an in-vitro manner through the RT-PCR technique. NHA cell line served as a control in the present study, the other 4 cell lines were tumorous cell lines. It was observed that in comparison with the NHA cell line, the tumorous cell lines expressed LINC01433, MYLK-AS1, and LNCOG in a higher ratio, supporting our bioinformatic results (Figure 11).

## Discussion

Considerable attention has been devoted to glioblastoma (GBM), adult-onset Considerable attention has been devoted to glioblastoma, a malignancy affecting the brain, with recent investigations focusing on the involvement of long non-coding RNAs (lncRNAs) in its pathogenesis 27. LncRNAs, a distinctive class of RNA molecules exceeding 200 nucleotides in length, exhibit structural characteristics analogous to mRNAs. Despite their lack of protein-coding capacity, lncRNAs display greater functional versatility and tissue specificity than mRNAs 28. These molecules exert pivotal regulatory roles and govern diverse cellular processes implicated in the development and progression of glioblastoma, including angiogenesis, drug resistance, invasion, metastasis, apoptosis, and proliferation 29. Altered expression of specific lncRNAs in glioblastoma has been associated with histological differentiation, and malignancy indices, and identified as potential biomarkers for assessing the grade, prognosis, and treatment resistance of glioblastoma 30. Furthermore, lncRNAs intricately participate in essential signaling pathways and molecular mechanisms underlying glioblastoma, such as the PI3K/AKT/mTOR pathway, Wnt/β-catenin pathway, and epithelial-mesenchymal transition (EMT) 30. The exploration of the intricate interplay between lncRNAs and glioblastoma demonstrates an exciting promise for unraveling underlying molecular mechanisms, as well as identifying novel diagnostic markers and therapeutic targets 31. However, a comprehensive research endeavor is imperative to achieve a profound understanding of the specific functional roles and clinical implications of lncRNAs in the context of glioblastoma.

Sphingolipids possess dual identities, serving as pivotal structural constituents of the cell membrane surface, thereby influencing the stability of the intracellular milieu and the activity of key signaling molecules. Additionally, sphingolipids exert significant regulatory influence as major lipid components of the myelin sheath surrounding nerve axons. Consequently, a thorough exploration of sphingolipid metabolic processes holds the potential to inform future therapeutic strategies for glioblastoma 32, 33. Leveraging comprehensive transcriptome sequencing data, our study reinforces the notion that unraveling the biological significance of sphingolipid metabolic pathways could serve as a compelling driving force in the current therapeutic landscape of glioblastoma. Our investigation validates 17 lncRNAs involved in sphingolipid metabolism as novel predictive biomarkers for glioblastoma. Among these 17 genes, PAXIP1-AS2, MYLK-AS1, LINC01433, LNCOG, FOXD3-AS1, TPRG1-AS1, HOXD-AS2, SNAI3-AS1, SOX21-AS1 have already undergone extensive scrutiny within the realm of cancer research. Notably, FOXD3-AS1 not only exerts oncogenic effects through its influence on the transcriptional regulation of FOXD3 but also impacts the resistance to temozolomide (TMZ) in glioblastoma, thus affecting the long-term prognosis of patients 34, 35. Overexpression of HOXD-AS2 is indicative of an unfavorable prognosis in glioma patients, and its upregulation promotes resistance to temozolomide, enhances MGMT expression, and reinforces the invasiveness of glioblastoma cells 36, 37. Recent investigations have also established a close association between HOXD-AS2 and the process of CTCF with cohesin binding. Diminishing its expression level leads to the silencing of other genes within the HOXD locus, thereby mediating glioma cell death 38. Conversely, SOX21-AS1 likely influences miR-144-3p through a competing endogenous RNA (ceRNA) mechanism to regulate the Wnt/β-connexin pathway, exerting a pro-carcinogenic effect 39. Our study collectively demonstrates the diagnostic efficacy of these 17 genes, supported by evidence from column line graphs and the nomogram. Individually, these 17 lncRNAs undeniably hold potential as robust markers (individually or in combination) for glioblastoma.

Comparative to preceding investigations, exploratory models based on sphingolipid metabolism have demonstrated a marginal advantage, as corroborated by an AUC value of 0.889. This discovery further bolsters the proposition that the sphingolipid metabolic pathway harbors considerable potential within the therapeutic paradigm of glioblastoma 40, 41. Indeed, efforts to exploit the prognostic diagnostic value of sphingolipid metabolism-related genes in diverse tumors, namely, to achieve translational medicine value with the aid of the exploitation of relevant models, have always been made, for example, Pei et al. established a novel prognostic model composed of seven SRGs by incorporating the transcriptomic as well as the single-cell data from datasets such as TCGA, GEO, among others, and it reflected well the level of immune infiltration in patients with breast cancer and the level of immune checkpoint changes 42. In addition, sphingolipid metabolism-related genes have different roles in the tumor microenvironment of patients with oral cancer, renal cancer, hepatocellular carcinoma, and osteosarcoma, whichever is the same as the biological background information mentioned above, sphingolipid metabolism is broadly involved in the development of carcinogenesis and may be a distinctive label at the pan-cancer level 43-45. However, the diagnostic efficacy of the current model of LncRNA in the GBM paradigm is superior to all of the above, and we tentatively attribute this discrepancy to sphingolipids themselves as a unique neurologically involved protein.

Moreover, the patient cohort derived from existing models, assessed by the Risk Score, holds promise in the field of translational medicine. Upon juxtaposing our study population with patients afflicted by G2 and G3 grade tumors, a notable reduction in prognostic levels was observed among the high-risk group. This observation, coupled with the overarching trend of escalating disease malignancy, implies a plausible association between sphingolipid metabolic pathways and the progression of glioblastoma. It is worth noting that the current Risk Score effectively stratifies the glioblastoma population not solely based on PCA and tSNE outcomes, but also by taking into account the distinct characteristics of both higher and lower-risk groups. Pathway enrichment analysis conducted on the high-risk cohort accentuates the activation of the Amino sugar and nucleotide sugar metabolism pathway. Extensive studies have duly documented the fundamental role played by nucleotide sugar synthesis in governing nutritional sensing, stress responses, tumor cell growth, and drug resistance, potentially contributing to the observed high-risk profile 46. Furthermore, the activation of the cell cycle, a widely acknowledged hallmark of tumor malignancy, is also manifest in the high-risk population 47, 48.

The divergence in population grouping based on the Risk Score appears to be more conspicuous when considering the context of the immune microenvironment. Although existing studies of LncRNAs in the context of GBM immunotherapy are more limited, our current study seems to furnish a referenceable paradigm for immunotherapy relying on non-coding RNAs. Our findings indicate elevated StromalScore, ImmuneScore, and ESTIMATEScore values within the high-risk groups as compared to the low-risk groups. Notably, these elevated indicators are associated with heightened infiltration of immune cells. Integrating these findings with our analysis, we posit that the heightened sphingolipid metabolism is concomitant with enhanced immune cell infiltration, which, within the framework of escalated glioma malignancy, may suggest the potential for immunotherapy approaches. This hypothesis builds upon prior investigations into the tumor immune microenvironment 49, 50. To shed further light on this matter, we conducted a meticulous characterization of immune cell composition within the immune microenvironment across diverse groups. Our findings unveil a relatively substantial degree of infiltration by macrophages and neutrophils in the high-risk score group. This dual effect of macrophage and neutrophil infiltration poses challenges to the long-term prognosis of immunotherapy, introducing a degree of uncertainty to ongoing research 51-53. However, the activation of NK cells is lower in the high-risk population, which provides insights into the inferior outcomes observed within this group. Moreover, disparities in immune function were observed between the two groups, with pivotal immune pathways, including immune checkpoints, CCR, pro-inflammatory response, and MHC-I, exhibiting higher levels of activation in the high-risk group. This suggests that the survival prognosis within the high-risk group is accompanied by an immune response 54. These results bolster our existing conclusions and indicate that our signature may offer valuable insights for future studies on the immune microenvironment of tumors. Prospecting the immune microenvironment holds translational medical value for the application of immunotherapy, which is of utmost importance given the expanding population undergoing immunotherapy, often failing to meet optimal therapeutic expectations. Hence, the identification of a range of biomarkers to predict the efficacy of immune checkpoint inhibitors becomes imperative 55-58.

Tumor mutational burden (TMB), along with its associated synchronous response to neoantigens, emerges as a robust and reliable indicator for monitoring the efficacy of immune checkpoint inhibitors (ICIs) 59-61. Prior studies on glioblastoma (GBM) have established a consensus, indicating that elevated TMB levels are intricately linked to diminished overall survival, primarily influenced by the extent of infiltration of CD8+ T cells and macrophages. Our investigation substantiates these findings, providing compelling evidence that heightened TMB levels in populations stratified based on sphingolipid metabolism levels—both in high-risk and low-risk categories—are significantly associated with a shortened prognosis. This groundbreaking revelation paves the way for innovative deliberations in future cancer immunotherapy, potentially fostering promising synergistic therapeutic approaches that integrate chemotherapy and ICIs, with a focus on metabolic pathways 62, 63. By shifting the emphasis of our study to genetic variations, we made a noteworthy observation: a notable frequency of mutations in the IDH1 gene within the low-risk populations. This observation aligns harmoniously with previous conclusions, further affirming the reliability and validity of our current model. Furthermore, it establishes the groundwork for a fresh paradigm in immunotherapy, capitalizing on the consensus derived from prior research, which identifies a significant correlation between IDH1 and TMB 64. To achieve this goal, we diligently pursued the establishment of connections with specific chemotherapeutic and immunotherapeutic agents, thereby fortifying and bolstering the strength of our conclusions.

## Conclusion

To recapitulate, our exhaustive inquiry accentuates the versatile applicability of the sphingolipid metabolic pathway as an autonomous diagnostic and prognostic marker in glioma patients. Furthermore, we amalgamate a plethora of substantiated evidence concerning immune infiltration and genetic mutations, thereby fortifying the proposition that sphingolipid metabolism could function as a pivotal determinant in the realm of immunotherapeutic interventions.
